# Dose-escalation, tolerability, and efficacy of intratumoral and subcutaneous injection of hemagglutinating virus of Japan envelope (HVJ-E) against chemotherapy-resistant malignant pleural mesothelioma: a clinical trial

**DOI:** 10.1007/s00262-024-03815-1

**Published:** 2024-10-03

**Authors:** Kazuma Sakura, Muneyoshi Kuroyama, Yasushi Shintani, Soichiro Funaki, Shinji Atagi, Yoshihisa Kadota, Kozo Kuribayashi, Takashi Kijima, Takashi Nakano, Toshihiro Nakajima, Masao Sasai, Meinoshin Okumura, Yasufumi Kaneda

**Affiliations:** 1 Respiratory Center, Osaka University Hospital, Suita, 5650871 Japan; 2 Department of Surgery, Osaka University Graduate School of Medicine, Suita, 5650871 Japan; 3 Department of Respiratory Medicine and Clinical Immunology, Osaka University Graduate School of Medicine, Suita, 5650871 Japan; 4 Department of Thoracic Oncology, National Hospital Organization, Kinki-Chuo Chest Medical Center, Sakai, 5918555 Japan; 5 Department of Thoracic Surgery, Osaka Habikino Medical Center, Habikino, 5838588 Japan; 6 Division of Respiratory Medicine, Department of Internal Medicine, Hyogo Medicine University, Nishinomiya, 6638501 Japan; 7 Division of Respiratory Medicine, Otemae Hospital, Osaka, 5400008 Japan; 8 Immunomedicine Inc., Osaka, 5410051 Japan; 9 Department of Medical Innovation, Osaka University Hospital, Suita, 5650871 Japan; 10 Department of Respiratory Surgery, National Hospital Organization, National Toneyama Hospital, Toyonaka, 5608552 Japan; 11 Administration Bureau, Osaka University, Suita, 5650871 Japan; 12 Present Address: Department of thoracic surgery, Hyogo Medical University, Nishinomiya, 6638501 Japan

**Keywords:** Malignant pleural mesothelioma;, Hemagglutinating virus of Japan envelope (HVJ-E), Phase I, Clinical trial

## Abstract

The hemagglutinating virus of Japan envelope (HVJ-E) is an inactivated Sendai virus particle with antitumor effect and inducing antitumor immunity. However, its dosage and efficacy have not been verified. We conducted a phase I clinical study on chemotherapy-resistant malignant pleural mesothelioma (MPM) aiming to determine the recommended dosage for a phase II study through dose-limiting toxicity and evaluate HVJ-E’s preliminary efficacy. HVJ-E was administered intratumorally and subcutaneously to the patients with chemotherapy-resistant MPM. While no serious adverse events occurred, known adverse events of HVJ-E were observed. In the preliminary antitumor efficacy using modified response evaluation criteria in solid tumors (RECIST) criteria, three low-dose patients exhibited progressive disease, while all high-dose patients achieved stable disease, yielding disease control rates (DCRs) of 0% and 100%, respectively. Furthermore, the dose-dependent effect of HVJ-E revealed on DCR modified by RECIST and the baseline changes in target lesion size (by CT and SUL-peak; *p* < 0.05). Comparing targeted lesions receiving intratumoral HVJ-E with non-injected ones, while no clear difference existed at the end of the study, follow-up cases suggested stronger antitumor effects with intratumoral administration. Our findings suggest that HVJ-E could be safely administered to patients with chemotherapy-resistant MPM at both study doses. HVJ-E exhibited some antitumor activity against chemotherapy-resistant MPM, and higher doses tended to have stronger antitumor effects than lower doses. Consequently, a phase II clinical trial with higher HVJ-E doses has been conducted for MPM treatment. Trial registration number: UMIN Clinical Trials Registry (#UMIN000019345).

**Supplementary Information:**

The online version contains supplementary material available at 10.1007/s00262-024-03815-1.

## Introduction

Malignant mesothelioma is a refractory tumor occurring primarily in the pleura and is rare compared to other malignancies, with an even lower incidence in other sites such as the peritoneum, pericardium, and tunica vaginalis [1]. Malignant pleural mesothelioma (MPM) accounts for the majority of malignant mesothelioma diagnosis, ranging from approximately 7 to 30% [2]. Globally, an estimated 30,870 individuals were diagnosed with mesothelioma, and 26,278 people died of mesothelioma worldwide in 2020 [3].

MPM is a refractory tumor associated with pleural effusion, dyspnea, and local pain. Median survival time and 1-year survival rate are 12.1 months and approximately 50%, respectively [4–6]. It is classified as epithelial (50–60%), biphasic (30–40%), or sarcomatoid (10%) based on pathomorphological findings, each with distinct prognostic implications [7]. The median survival times with treatment are 16.9 months, 13.1 months, and 5.5 months for the epithelial, biphasic, and sarcomatoid types, respectively, according to case reviews [4].

MPM is usually treated using multimodal approaches, including chemotherapy, immunotherapy, radiation therapy, and surgery. Chemotherapy (pemetrexed and cisplatin) is the basis for the treatment of mesothelioma and is performed as single or combined immunotherapy for inoperable cases and as neoadjuvant, intraoperative, or adjuvant chemotherapy for operative cases. [8].

The immune checkpoint inhibitors (ICIs) are one of the main methods for MPM treatment. Nivolumab was approved by the Japanese regulatory authorities in 2018 as a second-line treatment for MPM. Furthermore, nivolumab was approved the combination of nivolumab and ipilimumab as a first-line treatment for unresectable MPMs on October 2, 2020, in the USA, after clinical trials demonstrated a statistically significant improvement in overall survival (OS) in patients with unresectable MPMs treated with this combination therapy compared with patients treated with chemotherapy [9, 10].

ICIs are expected to be as effective for MPM treatment as they are for lung cancer management; however, concerns regarding curation still exist, because MPM is often already diagnosed in an advanced stage, and unlike lung cancer, it often presents as a disseminated tumor. Moreover, it has been considered difficult to fairly determine the effect of ICI on the survival of MPM patients recently, because real-world data on the efficacy of ICI show poor survival outcomes and more toxicity compared to clinical trial data, and concerns have been raised that inadequacies of each trial (selection criteria, frailty, and censoring patterns) affecting the conclusions obtained in previous trails [11, 12]. Therefore, in addition to lung cancer and other carcinomas, the development of new treatments to further improve the efficacy of MPM is highly desirable.

Hemagglutinating virus of Japan (HVJ) belongs to the paramyxovirus family, Paramyxoviridae, which causes parainfluenza in mice. Although HVJ can cause pneumonia in mice, it does not cause disease in humans because of specific differences in the host enzymes required for infection [13]. HVJ and HVJ whose internal RNA is fragmented artificially and inactivated (non-viral) HVJ (HVJ-E) possess unique characteristics in that they can induce cell fusion through the F and HN proteins in their outer membrane [14].

HVJ-E had antitumor activity in CT-26 and Balb/c mouse models of commensal cutaneous tumors. This antitumor effect involves the activation of dendritic cells and cytotoxic lymphocytes, generation of natural killer cells, and inhibition of regulatory T cells [15]. Furthermore, HVJ-E exhibits direct tumoricidal activity by inducing cell death via the RIG-I/MAVS pathway [16].

Virus-based cancer therapies, primarily investigated as virotherapy, focus on oncolytic virus therapy (OVT) [17]. Various viruses, such as herpes simplex virus type-1, measles virus, and other viruses, have been tested against mesothelioma [18–22]. Clinical trials have demonstrated that modified adenovirus, particularly adV/hIFN-α2b, significantly increased OS in the secondary treatment of mesothelioma, with chemotherapy [23]. The outcomes of other therapies are eagerly awaited. HVJ-E is virotherapy but is not classified as OVT. In OVT, specifications in isolated areas such as mesothelioma or bladder cancer make sense because of diffusion control, but the possibility of replication cannot be ruled out and requires strict control. In contrast, HVJ-E is more useful for clinical use because it is not replicable and can be used in general hospital wards. In addition, while most OV therapies for mesothelioma are injected as a solution into the pleural cavity, HVJ-E is only administered intratumorally or subcutaneously into the intercostal space, which has the advantage of causing fewer systemic effects.

The first human clinical trial of HVJ-E was conducted at Osaka University in 2009 in patients with advanced melanoma, followed by clinical trials in patients with advanced prostate cancer in 2011 [24]. In the clinical trials of HVJ-E in melanoma, and prostate cancer, HVJ-E induced the infiltration of immune cells into tumor tissue, suggesting that HVJ-E may have antitumor activity [25, 26]. The antitumor activity of HVJ-E against human mesothelioma was also studied in an in vivo orthotopic implant model. The results confirmed that HVJ-E had antitumor activity in a human mesothelioma orthotopic model compared with the control group [27]. Based on these promising preclinical data, we conducted a phase I clinical trial aimed at evaluating the tolerability and preliminary efficacy of HVJ-E in patients with chemotherapy-resistant pleural mesothelioma.

## Materials and methods

### Clinical research overview

This single-arm, open-label, single-center phase I/II clinical trial investigated the effect of intratumoral and subcutaneous HVJ-E administration in patients with chemotherapy-resistant MPM. This study was conducted at Osaka University Hospital, Japan, from 2015 to 2017, in accordance with the Declaration of Helsinki. The study protocol was approved by the Institutional Review Board, and written informed consent was obtained from all patients (IRB approval #157908). The study was registered in the UMIN Clinical Trials Registry (#UMIN000019345). Because this was a phase I study with safety as the primary endpoint, safety was evaluated in a 3 + 3 design, which is a common design for clinical trials of anticancer drugs. The incidence of dose limited tolerance (DLT) was assessed in three patients, and results of this assessment led to a maximum enrollment of six patients per dose. DLT was defined as any definite causal CTCAE v4.0 [28] grade 4 or higher hematological toxicity, grade 4 or higher onset of fever, or grade 3 or higher non-hematological toxicity (excluding fever) occurring, excluding grade 3 hematological toxicity. If febrile neutropenia is observed among grade 3 hematological toxicities, the efficacy and safety monitoring committee will consider whether or not to treat it as a DLT.

In a previous first-in-human clinical trial, dose escalation studies were conducted at doses of 3000 and 10,000 mNAU, suggesting that further dose escalation was feasible. Based on these results, a lower dose of 30,000 mNAU, which is three times the 10,000 mNAU dose, was established in this clinical trial. The higher dose of 60,000 mNAU was set based on the NOAEL (no observed adverse effect level) [29] obtained in the intermittent subcutaneous toxicity study conducted as a non-clinical safety study.

### Patients

Patients with chemotherapy-resistant MPM were included in this study. Chemotherapy-resistant MPM was defined as a progressive disease (PD) despite receiving first-line chemotherapy or second-line therapies such as FAK inhibitors or anti-CTLA-4 antibody therapy as an investigational treatment at the time of treatment. Injection criteria were set in the patient exclusion criteria (supplementary Table 1). The lesions with a thickness that was less than approximately 15 mm were difficult to inject and assess and therefore, were excluded as cases that the investigators deemed unsuitable for inclusion in this study for any reason. Some patients who participated in the trial underwent radiation therapy or surgery before becoming resistant to chemotherapy. Patients who were allergic to HVJ-E, according to the prick test, had brain metastases due to MPM, had interstitial pneumonia and pulmonary fibrosis, received chemotherapy within 6 weeks, received other experimental treatments, received immunotherapy for 4 weeks, or had an autoimmune disease or malignancy other than MPM within the past 5 years were excluded.

### HVJ-E preparation

HVJ-E was manufactured by Ishihara Sangyo Kaisha, Ltd. (Osaka, Japan). Irradiation of HVJ-E with propiolactone and UV light caused alkylation and fragmentation of the RNA genome. Subsequently, HVJ-E was purified by four-column chromatography steps, stabilized by lyophilization, and stored at 4 °C. The lyophilized HVJ-E was dissolved in distilled water for injection prior to administration [14].

### Treatment schedule

**Fig. 1:**
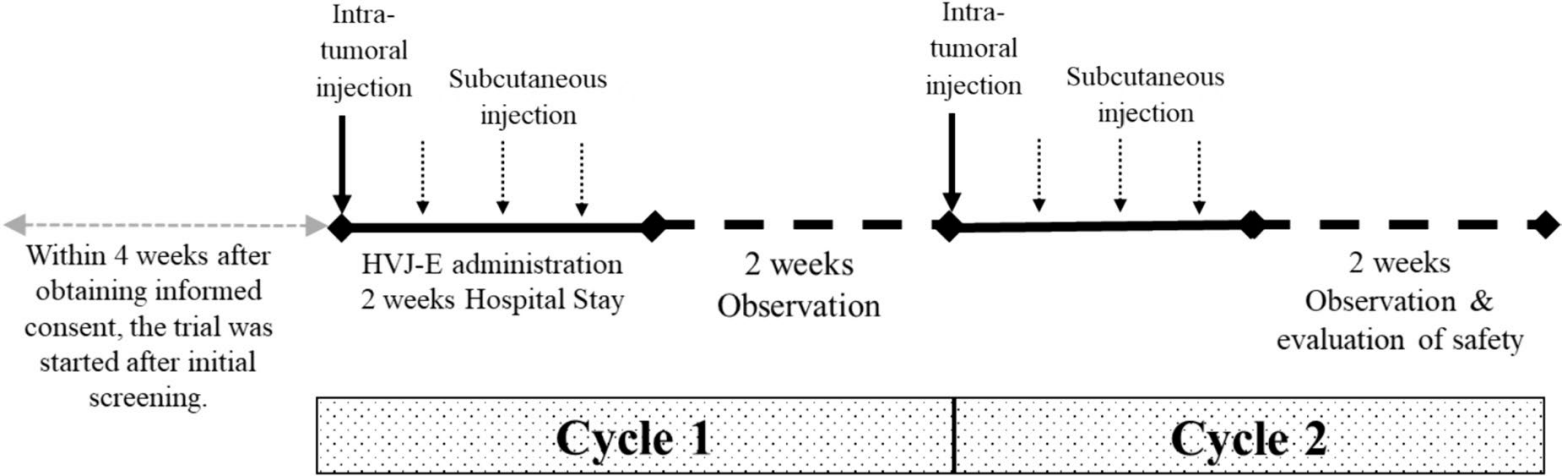
Clinical trial timeline of the study protocol

A 3 + 3 dose escalation design was used to determine the maximum tolerated dose of HVJ-E [30]. Under local anesthesia, patients with chemotherapy-resistant MPM were first directly injected with HVJ-E into the 18-fluorodeoxyglucose (FDG)-accumulated mesothelioma tissue by FDG-positron emission tomography (PET) screening. Using a 22-gauge cathelin needle (Terumo, Tokyo, Japan), a maximum of 1 mL per site was injected into the tumor through the intercostal space under ultrasound guidance (Arietta60, Hitachi-Aloka, Tokyo, Japan) on day 1. Before direct injection, tumor vessels and their firmness were examined using Doppler and elastography. HVJ-E was thereafter injected into the tumor to avoid the intercostal and tumor vessels. After the needle was removed, hemostasis was achieved, and no fluid reflux occurred. After injecting the HVJ-E solution, the patient’s condition (body temperature, blood pressure, absence of pneumothorax, and SpO2) was evaluated by chest radiography, and vital signs were monitored until the next morning. Subcutaneous injection of HVJ-E into the chest wall near the intratumoral injection site was performed on days 5, 8, and 12 in one cycle without anesthesia. Furthermore, eight HVJ-E injections were administered over two cycles (Fig. 1).

Patients with chemotherapy-resistant MPM first undergo direct injection of HVJ-E into the mesothelioma tissue guided with ultrasonography on day 1. Subsequently, subcutaneous injection of HVJ-E into the chest wall near the tumor was performed on days 5, 8, and 12 in one cycle. In total, eight HVJ-E injections are administered in two cycles. HVJ-E: Inactivated hemagglutinating virus of Japan envelope; MPM, malignant pleural mesothelioma.

### Safety and effectiveness assessment

Vital signs of the patients were monitored, and blood tests and chest radiography performed during the 14-day inpatient and outpatient visits of each cycle, as appropriate. Adverse events were graded using the National Cancer Institute Common Terminology Criteria for Adverse Events (version 4.0). Computed tomography (CT) and FDG-PET were performed at screening and on day 28 for preliminary assessment of efficacy.

### Statistical analysis

The sample size was based on previous clinical studies on HVJ-E in melanoma (#UMIN000002376) and castration-resistant prostate cancer (#UMIN000006142). The analysis population was defined as patients who completed the HVJ-E treatment. Characteristics of the registered patients and changes from baseline values (tumor reduction effect) were determined using *t*-tests and survival tests performed using log-rank tests.

## Results

### Registration status of patients with chemotherapy-resistant MPM for clinical trials

**Fig. 2:**
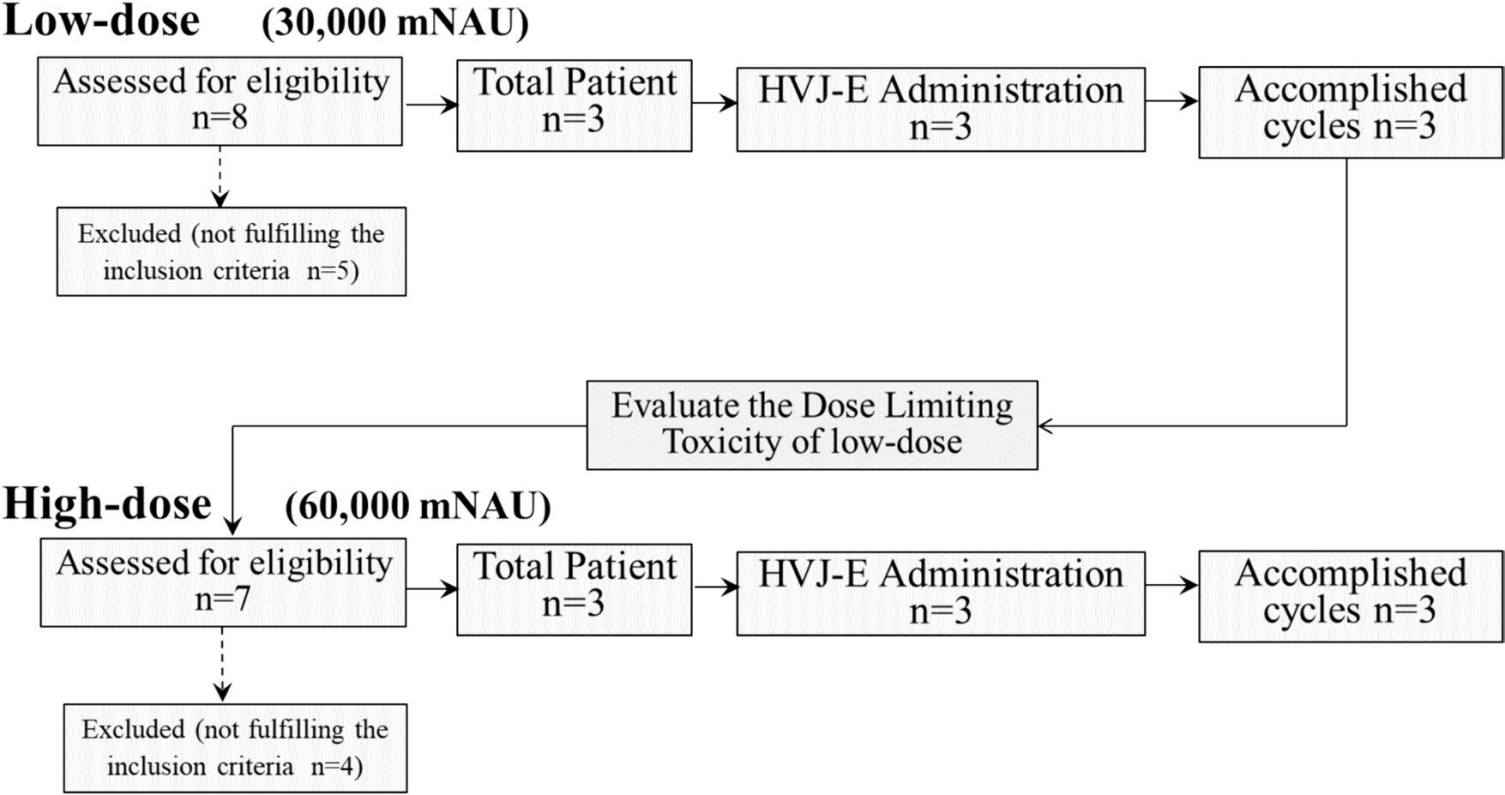
Diagram of patients with chemotherapy-resistant MPM who are enrolled in the low-dose (30,000 mNAU) or high-dose (60,000 mNAU) treatment groups

Eight patients were initially enrolled in the study; however, five were excluded based on exclusion criteria. Therefore, three patients were enrolled in a low-dose study to evaluate dose-limiting toxicity at low doses. No severe toxicity was observed at a lower dose, and patient recruitment proceeded to the next step of HVJ-E administration at a higher dose. Here, seven patients were enrolled, and four were excluded based on the exclusion criteria; hence, three patients were included in the high-dose study. Therefore, six patients were enrolled in this study to evaluate the safety and preliminary antitumor efficacy (Fig. 2).

Initially, eight patients are evaluated for enrollment in this study; however, five of them are excluded by the exclusion criteria. Three patients are enrolled in this study, and they are evaluated for the dose-limiting toxicity of HVJ-E administration at a low dose. No serious toxicity is observed at the low dose; thus, we proceed to the next step using a high dose of HVJ-E. Here, seven patients are evaluated for high doses, among whom four are excluded. The remaining three patients are, therefore, enrolled in the high-dose study, and this study is completed. HVJ-E: Inactivated hemagglutinating virus of Japan envelope; MPM, malignant pleural mesothelioma.

No significant differences existed in the characteristics of the recruited patients between the two HVJ-E dose groups in terms of age, disease stage, or time from the first visit to study entry (Table 1).

**Table 1:** Characteristics of patients with chemotherapy-resistant MPM treated with HVJ-E

		Low-dose group	High-dose group	Total
No. of patient		3	3	6
Age, years (range)		64.3 (61–68)	74.0 (66–81)	69.2 (61–81), *p > 0.1
Sex	M(%)	3 (100)	2 (66.7)	5 (83.3)
Primary side	rt.(%)	3 (100)	1 (33.3)	4 (66.7)
	lt	0	2(66.7)	2 (33.3)
Histology	Epithelial (%)	3 (100)	3 (100)	6 (100)
*Union for international cancer control (UICC) classification*				
T	T3(%)	2 (66.7)	3 (100)	5 (83.3)
	T4	1 (33.3)	0	1 (16.7)
N	N0	0	1 (33.3)	1 (16.7)
	N1	2 (66.7)	2 (66.7)	4 (66.7)
	N2	1 (33.3)	0	1 (16.7)
M	M0	3 (100)	3 (100)	6 (100)
Stage	Stage IB(%)	0	1 (33.3)	1 (16.7)
	IIIA	2 (66.7)	2 (66.7)	4 (66.7)
	IIIB	1 (33.3)	0	1 (16.7), *p > 0.1
*Previous therapies*				
Chemotherapy (%)		3 (100)	3 (100)	6 (100)
	Pemetrexed w/ or w/o Platinum	3 (100)	3 (100)	6 (100)
	Gemcitabine w/ or w/o CPT-11	1 (33.3)	3 (100)	4 (66.7)
Operation (%)		1 (33.3)	1 (33.3)	2 (33.3)
Radiation (%)		1 (33.3)	1 (33.3)	2 (33.3)
Others	FAK inhibitor (%)	0	2 (66.7)	2 (33.3)
	@CTLA-4 IgG2	0	2 (66.7)	2 (33.3)
Time from initial diag. to the start of clinical trial	(month)	23.2 (6.2–45.4)	36.9 (22.2–55.2)	30.1 (6.2–55.2), **p > 0.1

HVJ-E, inactivated hemagglutinating virus of Japan envelope; MPM, malignant pleural mesothelioma

### Safety of HVJ-E in patients with chemotherapy-resistant MPM

The adverse events and abnormal laboratory values are summarized in Table 2. Injection-related skin problems were the most common adverse events, and serious adverse events, including grade 3 events, were anemia in patients treated with a low-dose and increased levels of serum amylase in patients treated with a high dose. The most common adverse events were injection site erythema in both groups and skin induration and fever in the low-dose group (100.0% [3/3] of all patients), which were events expected from the route of administration and pharmacological effects, according to the previous clinical trials. Hypoxia, increased γ-glutamyl transferase, and increased blood alkaline phosphatase were observed in the low-dose group, and fever, puncture site pain, and skin induration were observed in 66.7% (2/3) of patients in the high-dose group. Grade 3 or higher adverse events included “increased amylase” levels in 16.7% (1/6) of patients. Adverse events were not considered to be related to the study drug, and the patients recovered promptly without treatment. Furthermore, no serious adverse events, deaths owing to adverse events, discontinuation, or adverse events leading to dose-limiting toxicity were observed. No changes, other than physiological changes in laboratory values or vital signs, were observed.

**Table 2:** Summary of adverse events and abnormal laboratory values in patients with MPM treated with HVJ-E

	Low dose (*n* = 3)		High dose (*n* = 3)		Total (*n* = 6)	
	No. of cases: grade, (%)		No. of cases: grade, (%)		No. of cases (%)	
	Adverse event	Casually related	Adverse event	Casually related	Adverse event	Casually related
*Symptoms and diseases*						
Erythema at injection site	3: G1 (100)	3: G1 (100)	3: G1 (100)	3: G1 (100)	6 (100)	6 (100)
Fever	3: G1 × 1, G2 × 2(100)	3: G1 × 1, G2 × 2 (100)	2: G1 × 1, G2 × 1,(66.7)	2: G1 × 1, G2 × 1,(66.7)	5 (83.3)	5 (83.3)
Skin sclerosis	3: G1 (100)	3: G1 (100)	2: G1 (66.7)	2: G1 (66.7)	5 (83.3)	5 (83.3)
Pain at the puncture site	0	0	2: G1 × 1, G2 × 1,(66.7)	2: G1 × 1, G2 × 1,(66.7)	2 (33.3)	2 (33.3)
Hypoxia	2: G2 (66.7)	2: G2 (66.7)	0	0	2 (33.3)	2 (33.3)
Pruritus	1: G1 (33.3)	1: G1 (33.3)	1: G1 (33.3)	0	2 (33.3)	1 (16.7)
Atrial fibrillation	1:G1 (33.3)	0	0	0	1 (16.7)	0
Dysphagia	0	0	1: G1 (33.3)	0	1 (16.7)	0
Nausea	1: G1 (33.3)	0	0	0	1 (16.7)	0
Vomit	1: G1 (33.3)	0	0	0	1 (16.7)	0
Bleeding at injection site	0	0	1: G1 (33.3)	1: G1 (33.3)	1 (16.7)	1 (16.7)
Pain at injection site	1: G1 (33.3)	1: G1 (33.3)	0	0	1 (16.7)	1 (16.7)
Itching at injection site	0	0	1: G1 (33.3)	1: G1 (33.3)	1 (16.7)	1 (16.7)
Small blisters at injection site	1: G1 (33.3)	1: G1 (33.3)	0	0	1 (16.7)	1 (16.7)
Nasal bleeding	0	0	1: G1 (33.3)	0	1 (16.7)	0
Contact dermatitis	1: G1 (33.3)	0	0	0	1 (16.7)	0
Subcutaneous bleeding	0	0	1: G1 (33.3)	1: G1 (33.3)	1 (16.7)	1 (16.7)
*Laboratory values*						
γ-glutamyl transferase increased	2: G1 (66.7)	1: G1 (33.3)	0	0	2 (33.3)	1 (16.7)
Alkaline phosphatase increased	2: G1 (66.7)	2: G1 (66.7)	0	0	2 (33.3)	2 (33.3)
Hypoalbuminemia	2: G2 (66.7)	0	0	0	2 (33.3)	0
Amylase increased	0	0	1: G3 (33.3)	0	1 (16.7)	0
Blood cholinesterase decreased	1: G1 (33.3)	1: G1 (33.3)	0	0	1 (16.7)	1 (16.7)
Haptoglobin increased	1: G1 (33.3)	1: G1 (33.3)	0	0	1 (16.7)	1 (16.7)
Platelet decrease	0	0	1: G1 (33.3)	0	1 (16.7)	0
Dehydration	1: G2 (33.3)	0	0	0	1 (16.7)	0
Hyperkalemia	1: G2 (33.3)	0	0	0	1 (16.7)	0
Erythema	0	0	1: G2 (33.3)	0	1 (16.7)	0
Anemia	1: G3 (33.3)	0	0	0	1 (16.7)	0
Lymphocytopenia	0	0	1: G3 (33.3)	0	1 (16.7)	0
Creatinine increased	0	0	1: G2 (33.3)	0	1 (16.7)	0
Hypocalcemia	1: G2 (33.3)	0	0	0	1 (16.7)	0

HVJ-E: inactivated hemagglutinating virus of Japan envelope; MPM, malignant pleural mesothelioma

Notably, no instances of feared skin dissemination of the tumor with reflux of the solution in case of intratumoral injection were detected during the observation period, extending at least 3 months.

### Efficacy of HVJ-E against patients with chemotherapy-resistant MPM

**Fig. 3:**
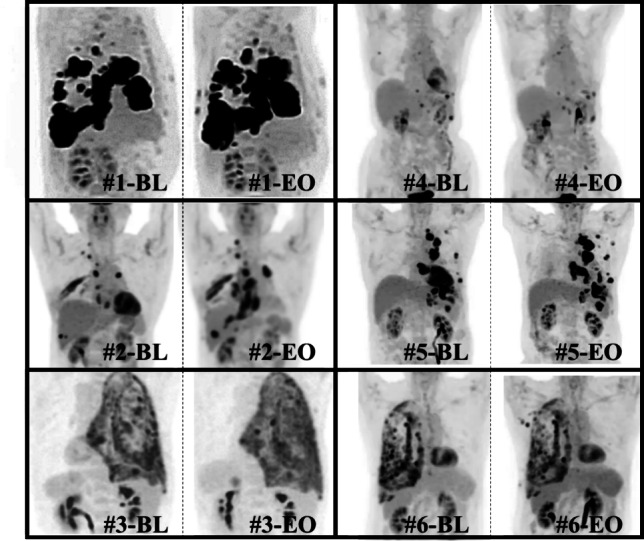
Whole tumor imaging using positron emission tomography (PET)/computed tomography (CT)

**Fig. 4:**
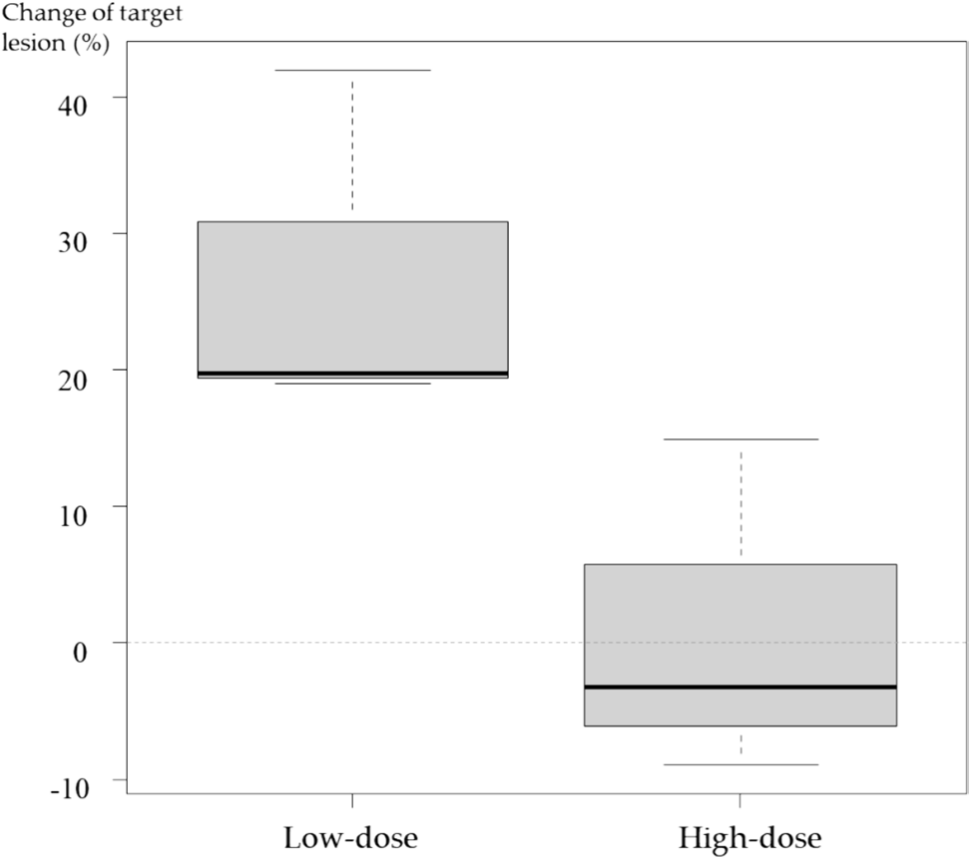
Dose dependency of HVJ-E with change of the target lesion by CT

**Fig. 5:**
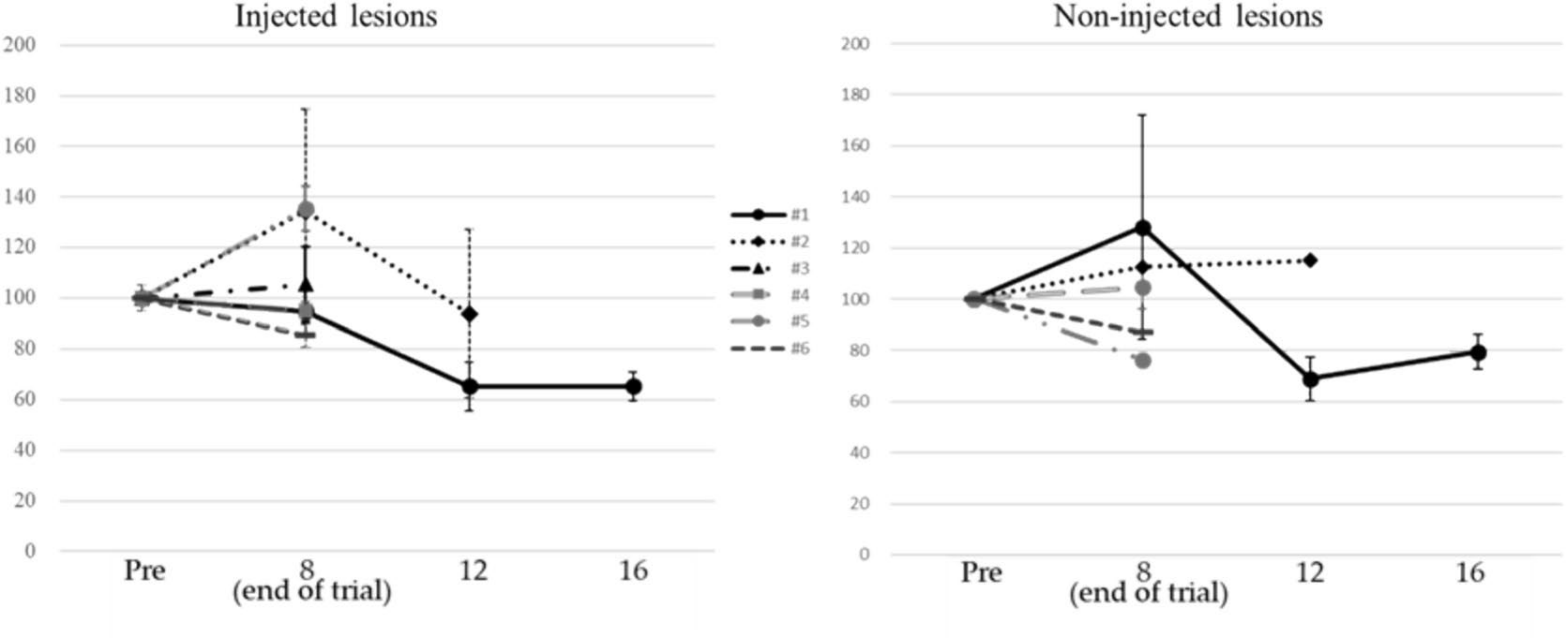
Difference in the reactivity of injected and non-injected lesions in the same cases evaluated by SUL-peak

According to the modified response evaluation criteria in solid tumors (RECIST) criteria [31], three patients achieved PD with the low dose, and three achieved stable disease (SD) with the high dose, with disease control rates (DCRs) of 0% and 100%, respectively (Table 3a). According to positron emission tomography response criteria in solid tumors (PERCIST) [32], all patients in both dose groups had stable metabolic disease (SMD) (Table 3b, Fig. 3). Dose dependence of HVJ-E was observed for baseline changes in target lesions on CT (*p* < 0.05) (Fig. 4) and RECIST assessment (CR, PR, SD, PD) at the standard uptake value (SUL) peak instead of CT (Table 3c). In a study of baseline changes in the SUL-peak between targeted lesions treated with intratumoral administration (intratumoral administration of HVJ-E with tumor cell death-inducing properties) and non-injected or non-targeted lesions, there was no clear difference at the end of the study. However, 1 month after the end of the study, the number of patients undergoing imaging was decreased, and although there was no significant difference between the two lesions, intratumoral and no intratumoral administration resulted in 20.4% and 8.0% antitumor efficacy, respectively. Two months after the end of the study, antitumor efficacy was observed in 34.7% and 20.4% of the two lesions treated with and without intratumoral administration, respectively, with a particularly strong trend toward antitumor efficacy with intratumoral administration (Fig. 5).

**Table 3:** Evaluation of antitumor efficacy

	(a) Evaluation of antitumor efficacy by CT according to the modified RECIST criteria						(b) Evaluation of antitumor efficacy by PERCIST criteria			(c) Evaluation of antitumor efficacy for each lesion by PET-CT (SUL-peak)							
Dose of HVJ-E	Case No	BL (cm)	BL 8 weeks after last injection (cm)	Evaluation	DCR		Evaluation	DCR		SUL-peak of each lesion				DCR			
					Each dose	Total case		Each dose	Total case	CMR	PMR	SMD	PMD	No. of target lesion	Each case	Each dose	Total case
Low dose (30,000 mNAU)	#1	171.6	205.4	PD	0% (0/3)	50% (3/6)	SMD	100% (3/3) (95% CI 29.2–100.0)	100% (6/6) (95% CI 54.1–100.0)	0	0	4	1	5	80.0%	76.9% (95% CI 45.4–95.9)	80.8% (95% CI 60.6–93.4)
	#2	72.75	86.54	PD			SMD			1	0	2	2	5	60.0%		
	#3	38.64	54.87	PD			SMD			0	0	3	0	3	100.0%		
High dose (60,000 mNAU)	#4	45.70	44.21	SD	100% (3/3)		SMD	100% (3/3) (95% CI 29.2–100.0)		0	0	5	0	5	100.0%	84.6% (95% CI 54.6–98.1)	
	#5	90.31	103.76	SD			SMD			0	0	2	2	4	50%		
	#6	141.88	129.17	SD			SMD			0	0	4	0	4	100.0%		

CI, confidence interval; CT, computed tomography; CMR, complete metabolic response; DCR, disease control rate; HVJ-E: inactivated hemagglutinating virus of Japan envelope; MPM, malignant pleural mesothelioma; SMD, stable metabolic disease

PET/CT scans are performed before and after the clinical trial (BL: Baseline, EO: end of observation) for each case (low-dose group; #1–#3 and high-dose group; #4–#6).

A significant difference is observed between low and high doses of HVJ-E with change of the target lesions, and the dose dependency of HVJ-E is disclosed. CT, computed tomography; HVJ-E: Inactivated hemagglutinating virus of Japan envelope.

In cases 1, 2, 4, 5, and 6, both injected and non-injected lesions can be evaluated, while in case 3, only injected lesions and not non-injected lesions as evaluation lesions are measured.

**Fig. 6:**
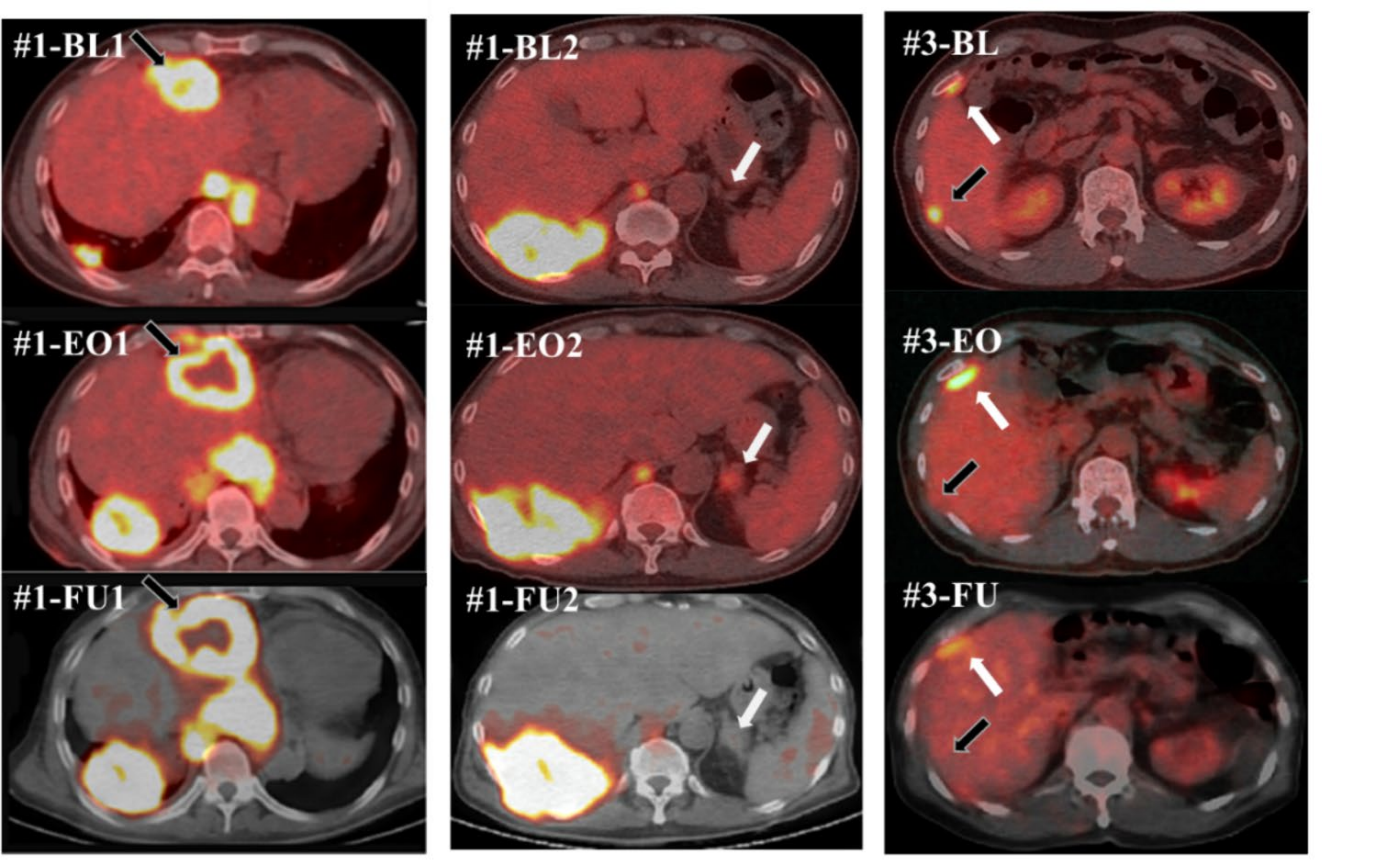
Representative PET/CT scan imaging of the tumors that respond well after being treated with HVJ-E in patients with chemotherapy-resistant MPM

In some cases, the accumulation of FDG at the sites of intratumoral administration and no injection was significantly reduced, and some tumors showed almost complete metabolic response (CMR) (Fig. 6).

#1-BL1 and #1-BL2 show the PET/CT scan images of case #1 at screening, #1-EO1 and #1-EO2 show the same lesion at the end of this trial, and #1-FU1 shows the scans 1 month after the end of this trial. #3-BL shows the PET findings of case #3 at screening, #3-EO shows the same lesion at the end of the trial, and #3-FU shows the scans 1 month after the end of the trial. The accumulation of FDG-PET in the lesion near the abdominal side is stronger in #1-EO2 but attenuated in #1-FU2. The gray and black arrows show the tumors injected with a low dose of HVJ-E. The white arrows show the non-injected site. BL: Baseline, EO, end of observation; FU: Follow-up; HVJ-E: Inactivated hemagglutinating virus of Japan envelope; MPM, malignant pleural mesothelioma.

## Discussion

In this study, we confirmed the safety and tolerability of HVJ-E at 30,000 and 60,000 mNAU in patients with chemotherapy-resistant pleural mesothelioma, since no serious adverse events or antitumor activity were observed. These findings align with those of previous clinical trials on melanoma and castration-resistant prostate cancer [24–26, 33].

Due to the same mechanism as that underlying skin dissemination after the biopsy of video-assisted thoracic surgery, which is often performed for diagnosis, concerns about local skin dissemination of lesions after intratumoral injection of HVJ-E [34] persist; however, no recurrence due to such local skin infiltration lesions was observed within the observation period. Although the RECIST results showed no response (Table 3a), the target lesion reduction rate based on DCR and FDG-PET/CT results was increased in a dose-dependent manner (Table 3b, Fig. 4). DCR is useful due to be set as a primary endpoint in the P-II study of MPM, and emphasized to be considered in the guidelines [35, 36]. PET/CT has also been reported to be useful imaging modality in determining treatment efficacy [37, 38]. Notably, the overall evaluation using the PERCIST assessment [39] revealed a 100% DCR (6/6) (Table 3b).

The results of the FDG-PET/CT assessments suggested that even low doses of HVJ-E could inhibit disease progression, whereas the results of the CT and FDG-PET/CT assessments suggested that greater inhibition of disease progression could be expected in the high-dose group. Based on these results, the recommended volume of HVJ-E for chemotherapy-resistant MPM is 60,000 mNAU. Detailed antitumor assessment of directly injected and non-injected lesions revealed no significant difference in local antitumor effect by SUL-peak (Fig. 5) and CT (supplementary Fig. 1).

However, 1 month after the study, the number of patients undergoing imaging was decreased; although no significant difference existed between the two lesions, the antitumor efficacy (SUL-peak baseline change) averaged 20.4% and 8.0% for intratumoral and non-intratumoral administration, respectively. In tumors that could be assessed 2 months after the study, antitumor effects were observed in 34.7% and 20.4% of the lesions treated with and without intratumoral administration, respectively, favoring intratumoral administration (Fig. 5). Notably, some cases showed significant FDG reduction at the intratumoral and non-injection sites, with some tumors showing values close to the CMR (Fig. 6).

In our study, the addition of HVJ-E to the prostate cancer cell line (LNCap) cells did not inhibit their proliferation; however, the addition of the mesothelioma cell lines MSTO-H211 and other human MPM cell lines to cell cultures significantly inhibited their proliferation. Moreover, intratumoral HVJ-E injection into murine mesothelioma subcutaneous tumor-bearing mice resulted in significant tumor shrinkage [27]. In this clinical study, direct HVJ-E injection into mesothelioma lesions resulted in a clear disappearance of mesothelioma cells on FDG-PET; however, not all lesions showed this response. In vivo, HVJ-E showed little direct cytotoxicity against the human prostate cell line LNCap, which may be due to differences in the expression of glycans that function as receptors for HVJ-E in tumor cells [40]. Therefore, it is important to investigate whether HVJ-E lesions have a more direct effect. However, in this clinical study, it was confirmed that the activation of antitumor immunity by HVJ-E produces antitumor effects, even in lesions that are not directly injected.

A comparison of Figs. 3 and 6, particularly Case #1, shows that the assessment of tumor progression before and after treatment does not always correlate with tumor volume and 2D images. (Cold images in the central region of the tumor were not correctly assessed.) While methods such as metabolic tumor volume (MTV) and total lesion glycolysis (TLG) have been explored for tumor volume evaluation in solid tumors, including this study, the results are not necessarily consistent with the assessment of the modified PERCIST criteria, as shown in the present study. Follow-up to investigate long-term prognosis and its correlation with antitumor effects is warranted.

The long-term prognosis was 44.9 months for the mean OS (mOS) after definitive diagnosis, which is relatively good considering that the prognosis for epithelial MPM with surgery (EPP, P/D) is approximately 18 months [41]. Furthermore, no dose dependence of HVJ-E exists in this case (supplementary Fig. 2). In contrast, the mOS of the four patients with inoperable MPM was 28.3 months, which is longer than the mOS in patients who received neoadjuvant chemotherapy but could not undergo surgery because of refusal for surgery, examination thoracotomy, or disease progression after neoadjuvant therapy [42]. The OS in all patients with inoperable disease over a 5-year period at the institute with the highest number of patients enrolled in this study was approximately 17 months [42], whereas the inoperable patients (four) were enrolled in this trial at 19.75 months after diagnosis, and it was guessed that they already had a PD. This suggests that HVJ-E may improve the prognosis even in patients who are not candidates for surgery (supplementary Fig. 3). Although no significant difference was observed between the two HVJ-E dose groups, a trend toward a longer prognosis exists in the high-dose group (Figure S3). In addition, the longer the time between confirmed diagnosis and study entry (with a threshold of 24 months), the more significantly HVJ-E improved the prognosis, even if the patient was refractory to chemotherapy (supplementary Fig. 4). This may be because patients after long-term treatment had smaller tumor volumes and relatively better general condition than patients after short-term treatment, even though they were enrolled in the study at the time when they were chemotherapy-resistant and had no further treatment options, and therefore had a better response to HVJ-E. While presented findings are, there some limitations in our study. First, it was a single-arm study focused on safety evaluation, and there was no concurrent control group. Other limitations were a single-center trial with a small number of patients, and pre-treatments were varied: chemotherapy, the presence or absence of surgery, or biological agents (FAK inhibitor, or anti-CTLA-4 antibody).

## Conclusions

HVJ-E could be safely administered to patients with chemotherapy-resistant MPM at both study doses. Another important finding is that higher doses of HVJ-E may have some inhibitory effect. These findings suggest that intratumor and serial subcutaneous injections of HVJ-E may possess antitumor efficacy with acceptable safety profile. Therefore, further investigation through larger, controlled clinical trials is warranted to comprehensively evaluate the efficacy and safety profile of the HVJ-E as a potential therapeutic option for this challenging disease.

## Patents

Patent cooperation treaty No. JP2017/039568 (WO2018/084185) is related to the content of this manuscript.

## Supplementary Information

Below is the link to the electronic supplementary material.

Supplementary file1 (DOCX 76 KB)

Supplementary file2 (DOCX 46 KB)

Supplementary file3 (DOCX 32 KB)

Supplementary file4 (DOCX 34 KB)

Supplementary file5 (DOCX 18 KB)

## Data Availability

The data supporting the findings of this study are available from the corresponding author, KS, upon reasonable request.
