# Supplementary material for: Dose-escalation, tolerability, and efficacy of intratumoral and subcutaneous injection of hemagglutinating virus of Japan envelope (HVJ-E) against chemotherapy-resistant malignant pleural mesothelioma: a clinical trial
Source: Cancer Immunol Immunother. 2024 Oct 3;73(12):243. doi: 10.1007/s00262-024-03815-1 (PMC11447170; doi:10.1007/s00262-024-03815-1)
Supplement: Supplementary file 1 — Supplementary file1 (DOCX 76 KB) [file 262_2024_3815_MOESM1_ESM.docx]

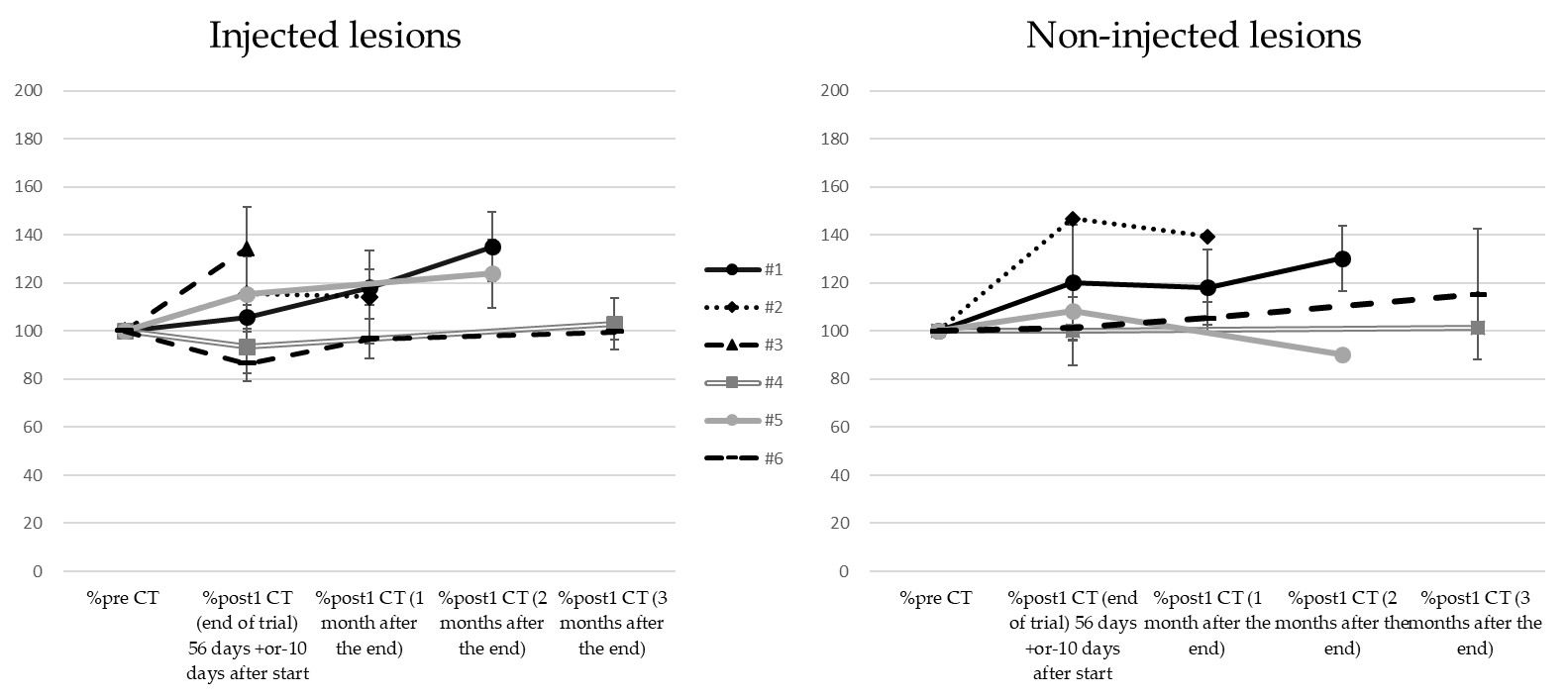


**Supplementary Figure 1. Difference in reactivity between injected and non-injected lesions evaluated by CT**

Difference in the reactivity of injected and non-injected leseions in the same cases evaluated by CT scan. Cases 1, 2, 4, 5 and 6 could evaluate both injected and non-injected lesions, while case 3 measured only injected lesions and not non-injected lesions as evaluation lesions.
