# Supplementary material for: Dose-escalation, tolerability, and efficacy of intratumoral and subcutaneous injection of hemagglutinating virus of Japan envelope (HVJ-E) against chemotherapy-resistant malignant pleural mesothelioma: a clinical trial
Source: Cancer Immunol Immunother. 2024 Oct 3;73(12):243. doi: 10.1007/s00262-024-03815-1 (PMC11447170; doi:10.1007/s00262-024-03815-1)
Supplement: Supplementary file 2 — Supplementary file2 (DOCX 46 KB) [file 262_2024_3815_MOESM2_ESM.docx]

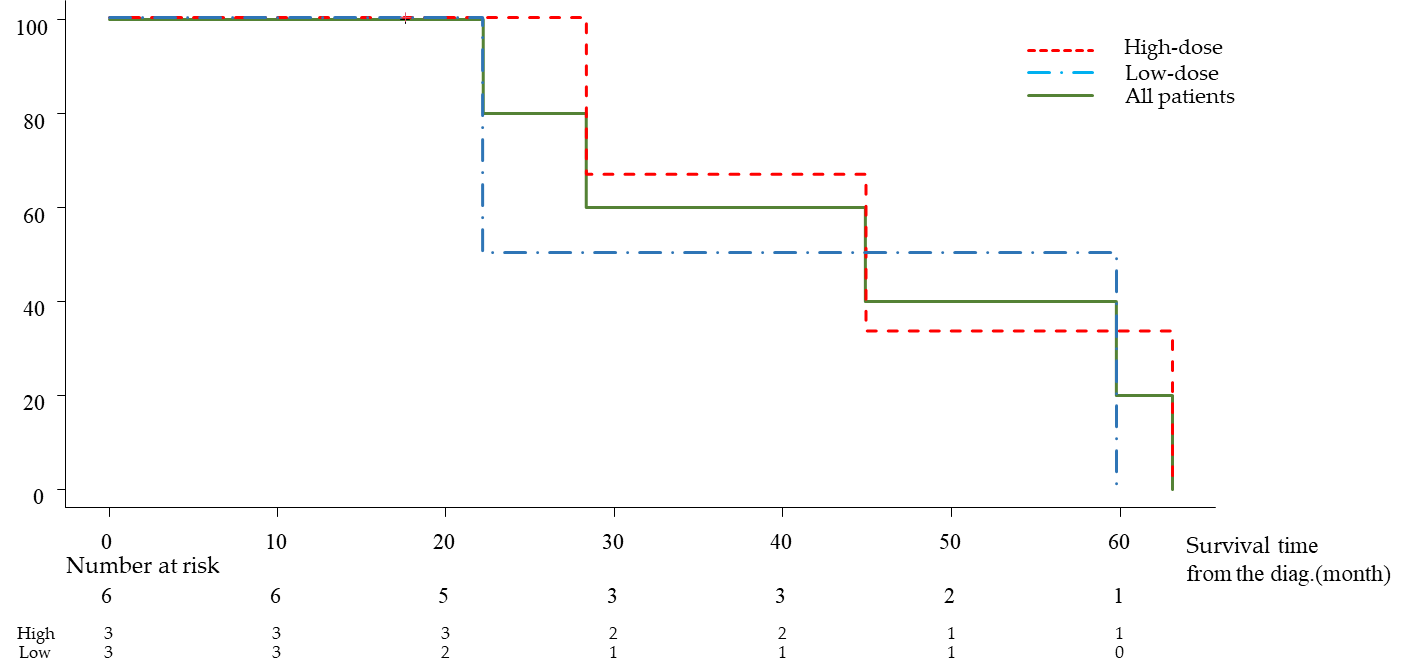


**Supplementary figure 2. Survival from definite diagnosis of patients with MPM treated with HVJ-E**

Survival of enrolled patients from final diagnosis of MPM. The median survival of all patients from a definitive diagnosis of MPM was 44.9 months. The median survival of the low-dose and high-dose groups was 44.9 and 44.1 months, respectively, and there was no significant difference between the two groups (*p* > 0.05).
