# Supplementary material for: Dose-escalation, tolerability, and efficacy of intratumoral and subcutaneous injection of hemagglutinating virus of Japan envelope (HVJ-E) against chemotherapy-resistant malignant pleural mesothelioma: a clinical trial
Source: Cancer Immunol Immunother. 2024 Oct 3;73(12):243. doi: 10.1007/s00262-024-03815-1 (PMC11447170; doi:10.1007/s00262-024-03815-1)
Supplement: Supplementary file 3 — Supplementary file3 (DOCX 32 KB) [file 262_2024_3815_MOESM3_ESM.docx]

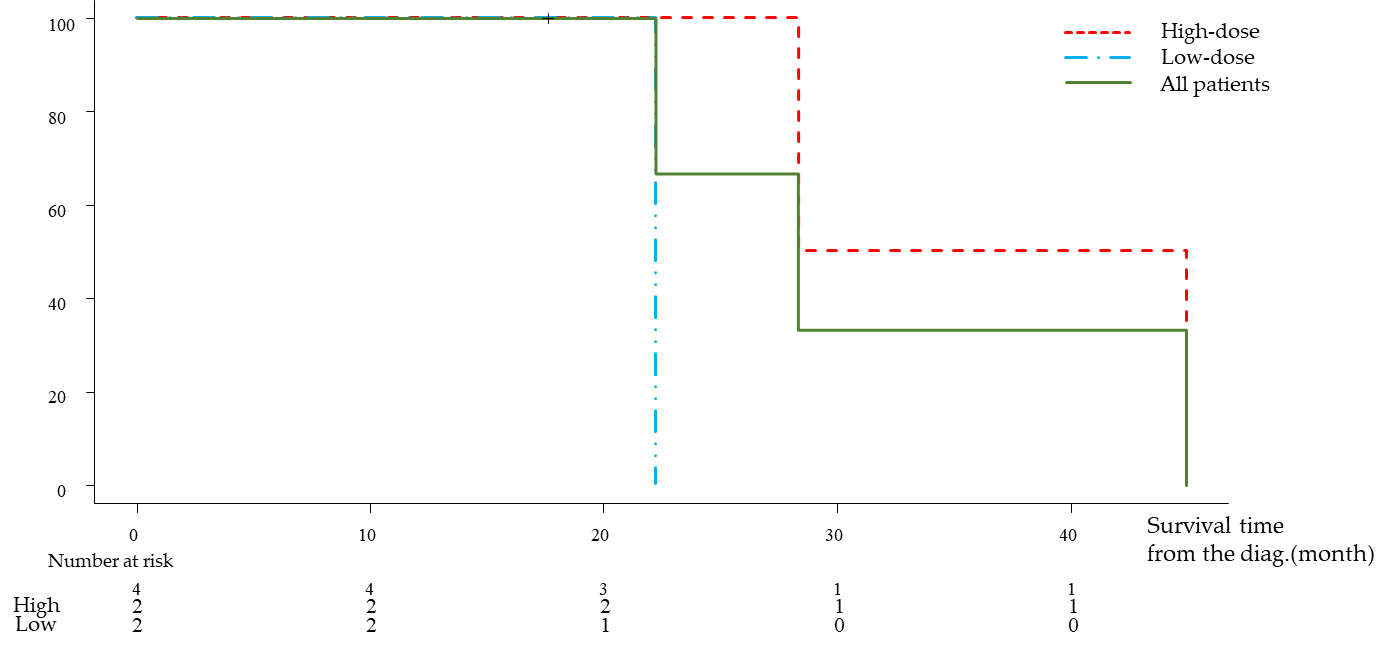


**Supplementary figure 3. Survival of patients with MPM treated with HVJ-E without operation**

Survival of enrolled patients treated without operation. The median survival of all patients from a definitive diagnosis of MPM was 28.3 months. The median survival of the low-dose and high-dose groups was 22.2 and 28.3 months, respectively, and there was no significant difference between the two groups (*p* > 0.05).
