# Supplementary material for: Dose-escalation, tolerability, and efficacy of intratumoral and subcutaneous injection of hemagglutinating virus of Japan envelope (HVJ-E) against chemotherapy-resistant malignant pleural mesothelioma: a clinical trial
Source: Cancer Immunol Immunother. 2024 Oct 3;73(12):243. doi: 10.1007/s00262-024-03815-1 (PMC11447170; doi:10.1007/s00262-024-03815-1)
Supplement: Supplementary file 4 — Supplementary file4 (DOCX 34 KB) [file 262_2024_3815_MOESM4_ESM.docx]

**
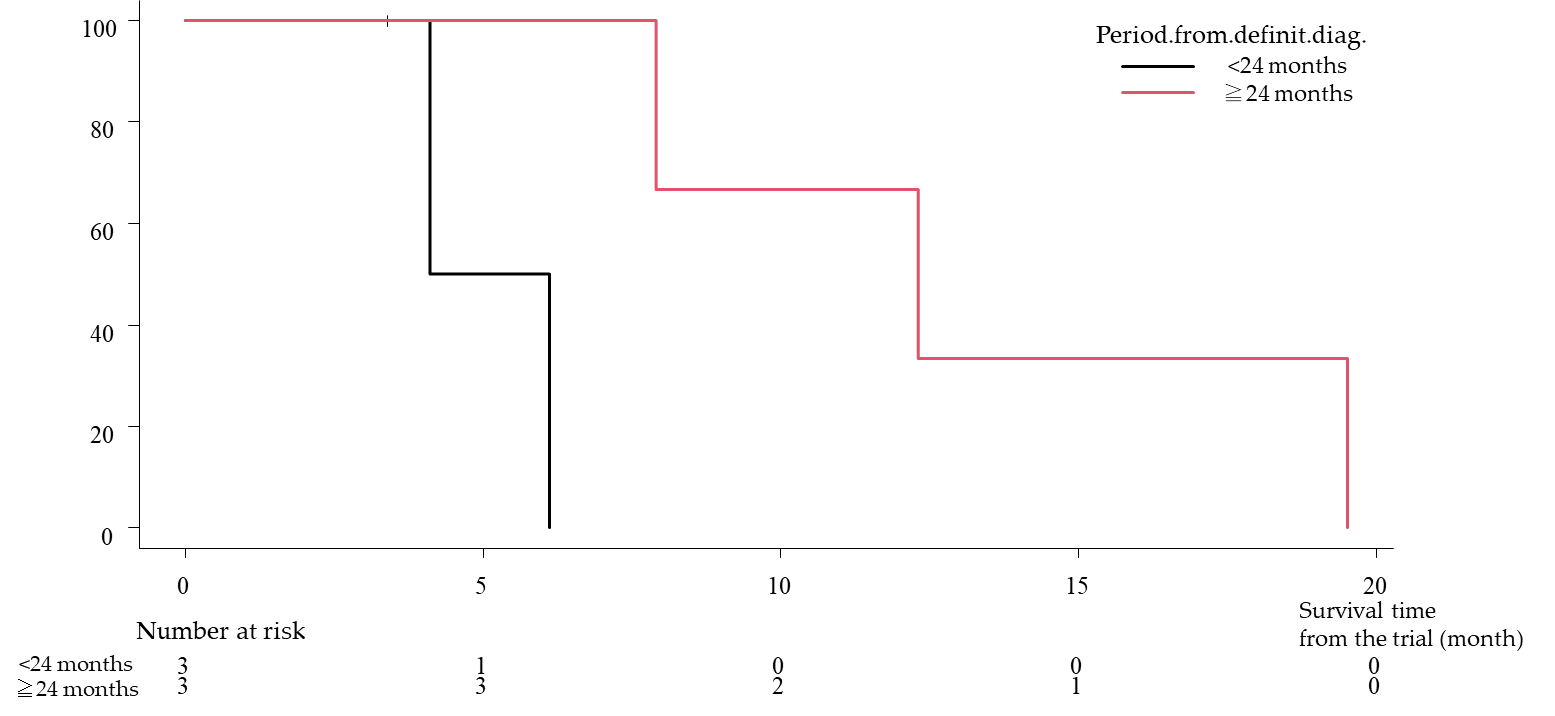
**

**Supplementary figure 4. Survival due to differences in the period from diagnosis to clinical trial enrollment**

Survival due to differences in time from diagnosis to clinical trial enrollment. The median survival of patients from a definite diagnosis to study entry of less than 24 months was 7.43 months, while the median survival of patients from a definite diagnosis of 24 months or more was significantly longer at 10.01 months (*p*< 0.05).
