# Supplementary material for: Dose-escalation, tolerability, and efficacy of intratumoral and subcutaneous injection of hemagglutinating virus of Japan envelope (HVJ-E) against chemotherapy-resistant malignant pleural mesothelioma: a clinical trial
Source: Cancer Immunol Immunother. 2024 Oct 3;73(12):243. doi: 10.1007/s00262-024-03815-1 (PMC11447170; doi:10.1007/s00262-024-03815-1)
Supplement: Supplementary file 5 — Supplementary file5 (DOCX 18 KB) [file 262_2024_3815_MOESM5_ESM.docx]

**Supplementary Table 1. Eligibility criteria**

| Inclusion criteria |
| --- |
| 1. Patients providing a written informed consent by voluntary agreement. 2. Age => 20 and =< 85 years old at the time of informed consent. 3. Have a diagnosis of malignant pleural mesothelioma as confirmed by histology. 4. Patients suffering from chemo-resistant malignant pleural mesothelioma, or patients without consecutive chemotherapy. - More than 6 weeks between the end date of the Chemotherapy and the registration date when the chemotherapy has been ineffective. 5. PaO2 >= 70 mmHg and SpO2 >= 93%. 6. Expected survival period is more than 8 weeks after planned start date of investigational product. 7. ECOG Performance Status 0 or 1. 8. Patients with lesions which can be detected by diagnostic imaging such as MRI or CT scan prior to treatment, and which can be administered with HVJ-E. 9. The marrow function, liver function and the kidney function must be kept as follows at the screening visit. 10. leukocyte >= 3,000/μL 11. neutrophil >= 1,500/μL 12. platelet >= 75,000/μL 13. hemoglobin >= 8.0 g/dL 14. AST =< 100 IU/L 15. ALT =< 100 IU/L 16. total bilirubin =< 2.5 mg/dL 17. serum creatinine =< 2.5 mg/dL |
| Exclusion criteria |
| 1. Have multiple brain metastases. 2. Positive result of the prick test of HVJ-E. 3. Have serious complications such as uncontrolled active infection. 4. Received systemic chemotherapy, radiotherapy or immunotherapy within 6 weeks before planned registration date. 5. Received another investigational product within 4 weeks before the informed consent. 6. Had a history of other malignancy, except for the relapse-free and metastasis-free for more than 5 years after the last treatment at the registration. 7. Have an interstitial pneumonia or fibrosis of the lung that need treatment. 8. Have an active autoimmune disease. 9. Receiving systemic administration of steroid formulation which restrains immunity response. 10. Had a history of the autologous or homogeneous organ or tissue transplantation (Receiving immunosuppressive medication). 11. PT (%) less than 10% of the lower limit of normal or APTT more than 1.5 times of the upper limit of normal of local reference range at the screening visit. 12. Pregnant or lactating women, except for the woman who discontinue to lactate (from the day of a written informed consent to the day after 30 days of discontinue the administration). In the case of woman, to conduct beta-HCG tests to confirm the presence or absence of pregnancy. 13. Positive result of the hepatitis B surface antigen, HCV antibody or HIV test at the screening visit. 14. Inappropriate to be enrolled in this study judged by the investigators, for any reason, including the possibility that the prognosis in the eligibility criteria may not be fulfilled. |
